# Improved Treatment Outcome Following the Use of a Wound Dressings in Cutaneous Leishmaniasis Lesions

**DOI:** 10.3390/pathogens13050416

**Published:** 2024-05-16

**Authors:** Pedro B. Borba, Jamile Lago, Tainã Lago, Mariana Araújo-Pereira, Artur T. L. Queiroz, Hernane S. Barud, Lucas P. Carvalho, Paulo R. L. Machado, Edgar M. Carvalho, Camila I. de Oliveira

**Affiliations:** 1 Instituto Gonçalo Moniz, FIOCRUZ, Salvador 40296-710, BA, Brazil; 2 Serviço de Imunologia, HUPES-UFBA, Salvador 40110-060, BA, Brazil; 3 Multinational Organization Network Sponsoring Translational and Epidemiological Research (MONSTER Initiative), Salvador 41720-200, BA, Brazil; 4 Instituto de Pesquisa Clínica e Translacional (IPCT), UniFTC, Salvador 41720-200, BA, Brazil; 5 Laboratório de BioPolímeros e Biomateriais, Uniara, Araraquara 14801-340, SP, Brazil; 6 INCT—Instituto de Investigação em Doenças Tropicais, Salvador 40110-040, BA, Brazil

**Keywords:** *Leishmania braziliensis*, skin lesion, meglumine antimoniate, topical therapy, wound dressing

## Abstract

Leishmaniasis, caused by *Leishmania* parasites, is a neglected tropical disease and Cutaneous Leishmaniasis (CL) is the most common form. Despite the associated toxicity and adverse effects, Meglumine antimoniate (MA) remains the first-choice treatment for CL in Brazil, pressing the need for the development of better alternatives. Bacterial NanoCellulose (BNC), a biocompatible nanomaterial, has unique properties regarding wound healing. In a previous study, we showed that use of topical BNC + systemic MA significantly increased the cure rate of CL patients, compared to treatment with MA alone. Herein, we performed a study comparing the combination of a wound dressing (BNC or placebo) plus systemic MA versus systemic MA alone, in CL caused by *Leishmania braziliensis*. We show that patients treated with the combination treatment (BNC or placebo) + MA showed improved cure rates and decreased need for rescue treatment, although differences compared to controls (systemic MA alone) were not significant. However, the overall time-to-cure was significantly lower in groups treated with the combination treatment (BNC+ systemic MA or placebo + systemic MA) in comparison to controls (MA alone), indicating that the use of a wound dressing improves CL treatment outcome. Assessment of the immune response in peripheral blood showed an overall downmodulation in the inflammatory landscape and a significant decrease in the production of IL-1a (*p* < 0.05) in patients treated with topical BNC + systemic MA. Our results show that the application of wound dressings to CL lesions can improve chemotherapy outcome in CL caused by *L. braziliensis*.

## 1. Introduction

Leishmaniasis is a neglected tropical disease caused by *Leishmania* parasites, with a major public health burden affecting 90 countries with an estimated one million new cases occurring each year [1]. In Brazil, *Leishmania braziliensis* is the main etiological agent of Cutaneous Leishmaniasis (CL), and between 2001 and 2020, more than 400,000 cases have been reported [2]. CL is the most common clinical form observed in individuals infected by *L. braziliensis,* but 3% of CL patients develop mucosal leishmaniasis concomitantly or years after the primary skin ulcer [3]. *L. braziliensis* may also cause disseminated leishmaniasis, a clinical manifestation in which parasites disseminate from the primary lesion, causing the appearance of multiple acneiform, nodular, and ulcerated lesions [4].

In Brazil, Meglumine antimoniate (MA) remains the first-choice treatment for CL despite the associated toxicity, adverse effects, need for parenteral administration, and increased reports of failure [3,5,6,7]. Even though Amphotericin B [8] and Miltefosine [9] have higher efficacy than MA, they are also associated with toxicity, teratogenic effects, and elevated cost. So far, the field has advanced towards the development of topical treatments based on single agents or combination therapy for CL but these advances are still under evaluation and have not been implemented in standard care [10]. However, another avenue of research is the possibility of using topical treatments that could have beneficial effects *per se*, such as wound healing properties, but that could also be used as delivery vehicles for chemotherapy, for example. In this scenario, Bacterial NanoCellulose (BNC) is a natural, non-toxic biocompatible nanomaterial [11], produced by different acetic acid bacteria strains that have been investigated for the purposes of wound dressing and drug delivery [12]. Among the properties of BNC properties, we highlight its three-dimensional reticulated network enabling a high capacity for moisture retention, while remaining permeable, flexible, and durable. A number of studies have shown that BNC has healing and tissue regenerative properties [13], reducing pain and bacterial infection [14], showcasing the applicability of this type of biomaterial for the treatment of chronic wounds, such as that observed in CL.

Based on these properties of BNC and on the inflammatory and tissue destructive nature of CL ulcers, we previously performed a proof-of-concept trial in which we evaluated the efficacy of a BNC wound dressing, applied topically to CL lesions, in combination with conventional systemic MA treatment, in CL caused by *L. braziliensis* [15]. In that study, CL patients treated with BNC dressings + systemic MA had a significantly higher cure rate at 60 days post initiation of treatment, compared to CL patients treated with MA alone. However, it remained to be determined whether the use of a wound dressing itself would have beneficial effects on the treatment outcome of CL lesions or whether this benefit was particular to BNC’s properties. Thus, we herein expanded on these initial findings, evaluating the efficacy of BNC wound dressings in comparison to the use of a placebo, in combination with systemic MA versus the use of systemic MA alone, in CL caused by *L. braziliensis*, in Brazil. We also evaluated the immune responses in these individuals, aiming to find possible immune markers associated with the outcome of a combination treatment (wound dressing plus systemic MA).

## 2. Materials and Methods

*Study Setting*. This study was conducted in the health post of Corte de Pedra, an endemic area for *L. braziliensis*, in Bahia, Brazil. The Brazilian Ministry of Health indicates systemic treatment for CL, routinely given as MA, administered IV for 20 days.

*Type of Study and Case Definition:* This was a randomized controlled trial to evaluate the efficacy of topical BNC wound dressings combined with intravenous MA in the treatment of CL. A total of 69 CL patients were admitted routinely from March 2021 to December 2023. Patients had only one ulcerated lesion measuring 1–5 cm in diameter, had an illness duration between >1 month and <3 months, and were 18–50 years of age. Skin biopsies were obtained from the edge of the ulcers with a 4mm punch biopsy, and CL diagnosis was performed by detection of *L. braziliensis* DNA by PCR [16]. The exclusion criteria were evidence of severe underlying disease (cardiac, renal, hepatic, or pulmonary), including serious infection other than CL; immunodeficiency or the presence of antibodies to the human immunodeficiency virus; pregnancy or lactation; and subjects with no ability to understand or no desire to give informed consent. All women of childbearing age underwent beta human chorionic gonadotropin tests to exclude pregnancy. Lesion biopsies were obtained with a 4 mm punch, on the border of the ulcer, from all patients.

*Group assignment and treatment*. We estimated that the rate of MA failure was 40% and that the rate of BNC cure was 80%. For a power of 80% and *p* < 0.05, we calculated 22 patients in each arm of this study. CL patients were randomized into three groups by simple randomization (www.randomization.com, accessed on 2 April 2021): BNC+MA (study group, 25 patients), PL+MA (placebo group, 21 patients), and MA (control group, 23 patients). Allocation rate was 1:1:1, and the random allocation sequence was implemented by sequential numbers. Both patients and physicians (including a dermatologist) attending to the patients were blinded after assignment to interventions. CL patients were randomized into three groups by simple randomization (www.randomization.com): BNC+MA (study group, 25 patients), PL+MA (placebo group, 21 patients), and MA (control group, 23 patients). The study group (BNC+MA) was treated with topical BNC membranes (kindly donated by Seven Biotecnologia-Nexfill, Londrina, PR, Brazil), placed onto the ulcerated lesion, and covered with Tegaderm Film (1624 W 3 M Health Care). BNC dressings were replaced three times a week for 3 weeks. Nexfill Biocellulose biocuratives are films composed of cellulose fibers with a nanometric structure, which are thin in thickness, non-toxic, and hypoallergenic. They have the same selective permeability of the skin, allowing normal sweating, preventing the escape of liquids, reducing the loss of electrolytes and proteins, and preventing the entry of microorganisms. The placebo group was treated with autoclaved gauze as a wound dressing, placed onto the ulcerated lesion, and covered with Tegaderm Film. Gauze dressings were replaced three times a week for 3 weeks. CL patients assigned to the study group (BNC) or placebo group (PL, autoclaved gauze) had their lesions cleaned with soap water before every application of the wound dressings (BNC or placebo). The cleaning and wound dressing procedure was conducted by a health care worker, at the Health Post. Cleaning and wound dressing procedures are part of routine care and were employed specifically for the purposes of this study. Patients in the study group (BNC) and in the placebo group (PL, autoclaved gauze) were simultaneously treated with systemic MA, administered intravenously at a dose of 20 mg/kg/day for 20 consecutive days (maximum daily dose of 1.215 mg). The control groups received MA alone. Patients were seen for follow up on days 30, 60, and 90 post initiation of treatment.

*Evaluation of the immune response.* Peripheral venous blood (25 mL) was collected from each patient at the time of diagnosis and 30 days after initiation of treatment to perform a complete blood count test; tests for aminotransferases (aspartate aminotransferase and alanine aminotransferase), urea, creatinine, sodium, and potassium; and immunologic studies. Peripheral Blood Mononuclear Cells (PBMCs) were obtained from heparinized venous blood layered over a Ficoll Hypaque gradient (GE Healthcare). Cells were washed and resuspended in RPMI 1640 medium (GIBCO) supplemented with 10% human AB serum, 100 IU/mL penicillin, and 100 μg/mL streptomycin (all Invitrogen). Cells (3 × 10^6^/mL) were plated in 24-well plates and stimulated with SLA (5 μg/mL) for 72 h at 37 °C and 5% CO_2_. Control cultures were left unstimulated. Cytokines were determined using MILLIPLEX MAP Human Cytokine/Chemokine Magnetic Bead Panel (Merck), according to manufacturer’s instructions, and results were expressed as pg/mL.

*Clinical outcomes*. The primary endpoint was cure defined as lesion healing with complete re-epithelialization of the lesions and no signs of raised borders at Day 90 after initiation of therapy. The secondary endpoints were initial cure at Day 60 and time-to-cure. Patients who failed therapy received a second course of MA for 30 days. Adverse effects (AEs) were graded according to the Common Terminology Criteria for Adverse Event v3.0 of the National Cancer Institute (https://ctep.cancer.gov/protocolDevelopment/electronic_applications/docs/ctcaev3.pdf, accessed on 20 April 2021).

### Statistical Analysis

Intention-to-treat analysis was performed to establish the cure rates. Continuous variables were characterized by median values and interquartile ranges (IQR) to indicate central tendency and dispersion. For categorical variables, frequency (number) and percentages were used for description. The comparison of categorical variables across different study groups was conducted using the Pearson’s chi-square test. Continuous variables were analyzed using the Mann–Whitney *U* test (comparison of two non-matching groups) or Kruskal–Wallis (comparison of three groups). Kaplan–Meier analysis using the Log-rank (Mantel–Cox) test was used to compare differences in time-to-cure among the three groups. For the heatmap illustration of immune profiles, the mean concentration for each immune mediator per patient group and time point (Day 0 and Day 30) were log-transformed. Statistical significance was determined for differences with *p*-values less than 0.05. All statistical analyses were carried out using R software, version 4.4.1, or GraphPad (Prism), version 10.1.1.

## 3. Results

A total of 69 CL patients were enrolled in the trial from May 2021 to January 2023. One patient from the placebo group only made use of two wound dressings. Two patients from the study group developed allergic reactions to MA. All three patients were considered as therapeutic failures and were excluded from the analyses (Supplemental Figure S1). The demographic and clinical characteristics of the patients were similar among the three groups (Table 1). Age ranged from 18 to 39 years old with a predominance of males in all three groups. Illness duration ranged from 30 to 45 days, and lymphadenopathy was prevalent (>56%) in all three groups (Table 1).

Following enrollment, CL patients were seen for follow up on day 30, 60, and 90 post initiation of treatment. Regarding therapeutic response, results showed that at D30, 3/25 patients in the study group (BNC+MA) and 3/21 patients in the placebo group (PL+MA) showed cure compared to none (0/23) in the control (MA alone) group (Table 2). At D60, cure increased to 11/25 patients in the study group (BNC+MA), 10/21 patients in the placebo group (PL+MA), and 7/23 in the control (MA alone) group. At D90, cure was observed in 17/25 patients in the study group (BNC+MA), 12/21 patients in the placebo group (PL+MA), and 13/23 in the control (MA alone) group. Thus, patients that made use of wound dressings (BNC or PL) + MA showed increased cure rates, at all three time points, compared to controls (MA alone). Although the cure rates were higher in patients that made use of wound dressings (BNC or PL) + MA, compared to controls (treated with MA alone), differences were not significant comparing the three groups or between groups (Table 2).

Rescue therapy was needed in 8/25 (32%) of patients in the study group (BNC+MA), 9/21 (43%) of patients in the placebo groups (PL +MA), and 13/23 (57%) of patients treated with MA only. Although the need for rescue therapy was lower in patients who made use of wound dressings, besides systemic MA, differences were not significant comparing the three groups or between groups (Table 2). When we evaluated time-to-cure, we observed a significant difference comparing the three groups (*p* = 0.025) (Table 2). Time-to-cure was significantly lower in patients treated with BNC+MA vs. MA alone (*p* = 0.019) and in patients treated with placebo (PL+MA) vs. MA alone (*p* = 0.026). Thus, the use of a wound dressing + systemic MA significantly decreased the time-to-cure in CL patients, reducing the median time-to-cure to <70 days in comparison to 105 days (MA alone) (Table 2).

**Figure 1:**
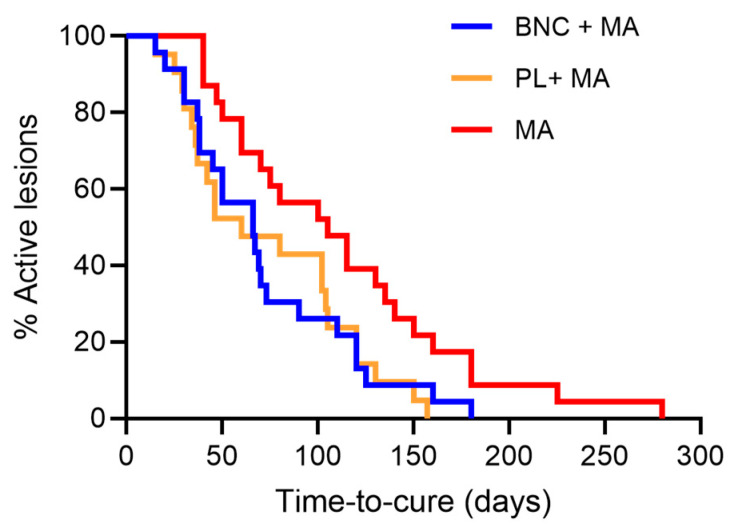
Kaplan–Meier curve comparing time-to-cure in the study group (BNC+MA), placebo group (PL+MA), and control group (MA alone). Time-to-cure is the number of days required for the complete resolution of ulcers, when signs of clinical activity such as inflammation or raised borders are no longer observed.

Overall, 8/25 (32%) patients in the study group (BNC+MA) remained with active ulcers compared to 9/21 in (43%) in the placebo group (PL+MA) and 13/23 patients (57%) in the control group (MA alone) [*p* = 0.018; Log-rank (Mantel–Cox) test] (Figure 1). Significant differences were also observed comparing BNC+MA vs. MA alone [*p* = 0.020; Log-rank (Mantel–Cox) test] and comparing PL+MA vs. MA alone [(*p* = 0.018); Log-rank (Mantel–Cox) test]. No significant differences were found comparing patients treated with BNC+MA vs. PL+MA.

**Figure 2:**
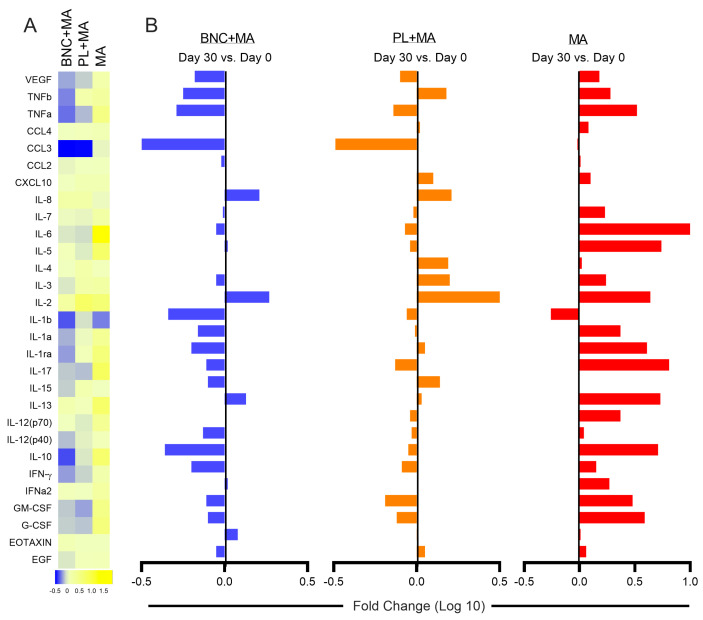
Use of wound dressings on CL lesions modulates the systemic immune response. (**A**) Data (mean PBMC supernatant concentration of each indicated mediator per patient group and time point) were log-transformed, and a heatmap was used to illustrate trends in data variation. (**B**) Fold differences for each mediator (D30–D0) were calculated, and log10 values (Supplementary Table S1) were plotted.

We also evaluated the cellular immune response in CL patients. Comparing cytokine production on Day 30 vs. Day 0 (before initiation of treatment), the production of TNFb, IL-1b, IL1a, IL1-ra, IL-15, and IL-10 was markedly reduced in patients treated with BNC + MA (Figure 2A). While patients treated with BNC+MA and PL+MA showed a reduction in the production of IL-1b, IL-1, IL-1ra, IL-15, and IFN-γ, this was not observed in patients in the control group (MA alone) (Figure 2B). In this group, most of the immune mediators evaluated, in particular inflammatory markers such as TNF, IL-6, IL-1a, IL-1ra, IL-12, IL-17, and IFN-γ, were upregulated (Figure 2B).

**Figure 3:**
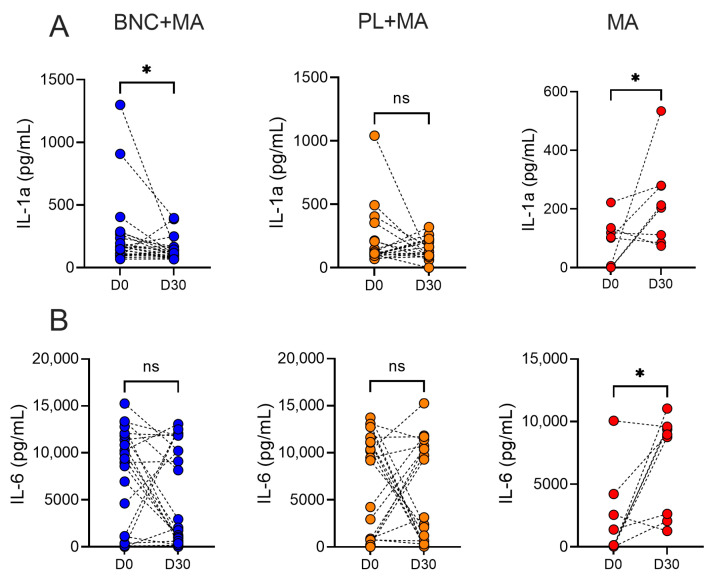
Cytokine production in CL patients. PBMCs were obtained from CL patients before (D0) and after therapy (D30). Cells were cultured with SLA for 72 h. Levels of IL-1a and IL-6 were determined by Luminex. Each symbol represents one CL patient. Blue (study group) (n = 25), orange (Placebo group, n = 21), and red (control group, n = 23). (**A**) IL-1a, (**B**) IL-6. * *p* < 0.05. Abbreviations: ns, not significant.

Only patients treated with BNC+MA showed a significant decrease in IL-1a levels, comparing D0 and D30, while an opposite result was observed in the control group (Figure 3A); on the other hand, only the control group (MA alone) showed significantly increased IL-6 production (Figure 3B), comparing D0 and D30. These results indicate that the use of a wound dressing downmodulates the inflammatory landscape at day 30 after treatment, especially IL-1a, accompanying the more favorable outcomes observed in these two groups (Table 2).

## 4. Discussion

In Brazil, MA remains the first-choice treatment for CL treatment despite its low cure rate. While Paromomycin and Imiquimod have demonstrated efficacy in CL caused by different species of *Leishmania* [10], there are no studies evaluating the efficacy of these medications in Brazil. In the absence of less toxic alternatives to treat CL, therapeutic modifications that can lessen the toxicity of systemic MA use could be promising. In this randomized clinical trial, we showed that the use of a wound dressing, in conjunction with systemic MA, enhanced the cure rate in CL caused by *L. braziliensis*, while decreasing the need for rescue therapy and the overall time-to-cure.

BNC has been evaluated in the context of chronic wound treatments, particularly given its ability to maintain local humidity and its action as a physical barrier, reducing external contamination and favoring the adsorption of inflammatory exudates. These characteristics have shown to accelerate the healing process, shortening lesion treatment time in burn wounds [17]. In another study with venous ulcers of the lower limbs, the use of BNC dressing significantly reduced the initial wound area, requiring fewer interventions and manipulation [18]. Based on these properties of BNC, we previously evaluated the efficacy of a topical intervention consisting of a BNC wound dressing + systemic MA for the treatment of CL lesions [15]. In that study, we found that patients treated with BNC+MA had a significantly higher cure rate at D60 compared to patients treated with MA alone. Herein, we build on this rationale of a combination treatment, comparing the topical use of BNC + systemic MA with use of a placebo, consisting of autoclaved gauze, + systemic MA. Control patients received systemic MA only. The use of a wound dressing (BNC or placebo) increased the cure rate in comparison to the use of MA alone; it also decreased the need for rescue therapy. Importantly, patients that used a wound dressing (BNC or placebo) showed a significantly lower time-to-cure, in comparison to those using MA alone. In the previous study [15] and herein, a routine of lesion cleaning with soap water was employed before every application of the wound dressings (BNC or placebo). This routine is not part of the standard care of CL patients and was not applied to patients treated with MA alone. Thus, regular lesion cleaning may account for the similar results observed in patients making use of the dressings (BNC or placebo). CL lesion microbial profiling showed the abundance of *Staphylococcus* sp. in CL lesions [19], and bacterial burden is associated with delayed lesion healing and enhanced inflammatory response [20]. Thus, regular lesion cleaning followed by dressing may have inhibited microbial growth, leading the better therapy outcomes.

Systemic antibiotics given prior to pentavalent antimonial (Sodium Stibogluconate) treatment also had no effect upon long-term healing (>6 months) outcome [21]. In trials evaluating the efficacy of topical Paramomycin in CL, patients’ lesions were also cleaned with soap water and covered with sterile gauze [22,23], but the authors found that superior cure rates were only found upon use of Paramomycin, indicating that the cleaning procedure had no impact. Use of Paramomycin + Gentamicin or Paramomycin alone were equally effective indicating that concomitant use of an antibiotic did not have any added effect [22]. We speculate that the use of a topical leishmanicidal agent such as Paramomycin may overcome the possible effects of lesion cleaning and dressing. On the other hand, regular lesion cleaning and dressing may be beneficial when CL is treated systemically with MA, as seen in the present study.

Besides the enhanced time-to-cure observed in patients treated with wound dressings, another finding was the modulation of the immune response observed on D30, compared to D0. Application of the wound dressing (BNC or placebo) decreased the production of inflammatory mediators, a finding not observed in patients treated with MA alone. In particular, patients treated with topical BNC showed a marked decreased production of IL-1a and IL-1b. In experimental leishmaniasis, mice lacking IL-1a or IL-1b displayed delayed disease development and more attenuated systemic inflammatory responses [24], while IL-1b has been associated with immunopathology in human CL caused by *L. braziliensis* [25,26]. In fact, it has been suggested that therapies targeting the host’s immune response should be explored in the context of CL, a disease in which the excessive production of IFN-γ and TNF are associated with pathologic responses [27]. Along this line, the combination of Pentoxifylline, a TNF inhibitor, plus MA showed promising results in Mucosal Leishmaniasis patients [28]. In experimental *L. braziliensis* infection, use of an immunomodulator (Tofacitinib) reduced the expression of Granzyme B, protecting mice from severe disease, without altering effector T cell responses [29]. Given our observation that wound dressing with BNC significantly reduced the production of IL-1, we speculate that the effects of BNC may possibly extend beyond its wound healing properties, as shown in diabetic foot ulcers [30], superficial burn injuries and skin graft sites [31], and chronic venous ulcers [18].

We are aware that a possible limitation of this study may have been the sample size, especially given the loss of follow up with three patients. Although an added benefit of topical use of BNC was not observed in comparison to placebo with regards to cure rate, we were able to show that a regular routine of lesion cleaning followed by application of a dressing significantly improved the time-to-cure of CL patients, in comparison to use of MA alone.

## 5. Conclusions

In conclusion, we build on our previous study [15] evaluating the combination treatment of standard MA plus a wound dressing. Here, we show that cleaning of CL lesions followed by use of a wound dressing reduced the time-to-cure significantly and decreased the production of inflammatory mediators, suggesting an overall beneficial effect of this procedure. Given that these effects were observed irrespective of the type of dressing, BNC, or autoclaved gauze (placebo), we suggest that this procedure could be adopted in the standard care of CL lesions caused by *L. braziliensis*. We believe that regular cleaning of lesions with soap water followed by lesion dressing with gauze has the potential to be implemented by health care workers and followed up on by patients, given the low cost and wide availability of such materials.

## Figures and Tables

**Table 1:** Demographical and baseline clinical characteristics of CL patients.

Characteristic	BNC+MA (N = 25)	PL+MA (N = 21)	MA (N = 23)	BNC+MA vs. PL+MA vs. MA	BNC+MA vs. PL+MA	MA vs. BNC+MA	MA vs. PL+MA
Age, Years ^&^	23 (18–40)	29 (21–38)	30 (22–35)	ns ^#^	ns ^¶^	ns ^¶^	ns ^¶^
Sex, Male, n/N (%)	18/25 (72%)	13/21 (62%)	16/23 (70%)	ns ^§^	ns ^§^	ns ^§^	ns ^§^
LST, mm^2^	196 (115–255)	225 (155–225)	180 (120–225)	ns ^#^	ns ^¶^	ns ^¶^	ns ^¶^
Illness duration, days ^&^	30(30–38)	30 (30–43)	30 (30–45)	ns ^#^	ns ^¶^	ns ^¶^	ns ^¶^
Size of largest lesion, mm^2 &^	264 (154–536)	240 (100–498)	300 (220–480)	ns ^#^	ns ^¶^	ns ^¶^	ns ^¶^
Ulcers on lower limbs, n/N (%)	18/25 (72%)	13/21 (62%)	20/23 (87%)	ns ^§^	ns ^§^	ns ^§^	ns ^§^
Lymphadenopathy, n/N (%)	19/25 (76%)	15/21 (71%)	13/23 (57%)	ns ^§^	ns ^§^	ns ^§^	ns ^§^

Abbreviations: BNC, Bacterial NanoCellulose; MA, Meglumine Antimoniate; PL, placebo; ns, not significant. ^&^ (median, interquartile range). ^§^ Pearson’s chi-squared test, ^¶^ Mann–Whitney test, ^#^ Kruskal–Wallis test.

**Table 2:** Response to therapy at D30, D60, and D90.

Response to Therapy, n/N (%)	BNC+MA (N = 25)	PL+MA (N = 21)	MA (N = 23)	BNC+MA vs. PL+MA vs. MA	BNC+MA vs. PL+MA	BNC+ MA vs. MA	PL+ MA vs. MA
Cure at D30	3/25 (12%)	3/21(14%)	0/23 (0%)	ns ^§^	ns ^§^	ns ^§^	ns ^§^
Cure at D60	11/25 (44%)	10/21(48%)	7/23 (30%)	ns ^§^	ns ^§^	ns ^§^	ns ^§^
Cure at D90	17/25 (68%)	12/21(57%)	10/23 (44%)	ns ^§^	ns ^§^	ns ^§^	ns ^§^
Rescue therapy	8/25 (32%)	9/21 (43%)	13/23 (57%)	ns ^§^	ns ^§^	ns ^§^	ns ^§^
Time-to-heal, days (median, IQR)	66 (38–110)	60 (35–113)	105 (60–150)	0.025 ^#^	ns ^¶^	0.019 ^¶^	0.026 ^¶^

Abbreviations: BNC, Bacterial NanoCellulose; PL, placebo; MA, Meglumine Antimoniate; ns, not significant. ^§^ Pearson’s chi-squared test; ^¶^ Mann–Whitney test; ^#^ Kruskal–Wallis test.

## Data Availability

The data presented in this study are available on request from the corresponding author.

## Supplementary Materials

The following supporting information can be downloaded at: https://www.mdpi.com/article/10.3390/pathogens13050416/s1, Figure S1: Study flowchart. Table S1. Per-protocol analysis of therapeutic response at D30, D60 and D90.
