# Supplementary material for: Improved Treatment Outcome Following the Use of a Wound Dressings in Cutaneous Leishmaniasis Lesions
Source: Pathogens. 2024 May 16;13(5):416. doi: 10.3390/pathogens13050416 (PMC11124396; doi:10.3390/pathogens13050416)
Supplement: Supplementary file 1 [file pathogens-13-00416-s001.zip › Table S1.pdf]

Supplementary Table S1. Per-protocol analysis of therapeutic response at D30, D60 and D90.

Response to therapy, n/N (%)	BNC+MA (N=23)	PL+MA (N=20)	MA (N=23)	BNC+MA *vs* PL+MA	BNC+ MA *vs* MA	PL+ MA vs MA
Cure at D30	3/23 (13%)	3/20 (15%)	0/23 (0%)	ns§	ns§	ns§
Cure at D60	11/23 (47,8%)	10/20 (50%)	7/23 (30.4%)	ns§	ns§	ns§
Cure at D90	17/23 (73,9%)	12/20 (60%)	10/23 (43.5%)	ns§		ns§
Rescue therapy	6/23 (26%)	8/20 (40%)	13/23 (56.5%)	ns§	0.0361§	ns§
Time-to-heal, days (median, IQR)	66.0 (38.0-110)	53(34.5-116)	105 (60-150)	ns¶	0.0185¶	0.0205¶

Abbreviation: IQR, interquartile range; ns, not significant.

§ Pearson's chi-squared test

¶ Mann-Whitney test
