# Supplementary figures and images for: Improved Treatment Outcome Following the Use of a Wound Dressings in Cutaneous Leishmaniasis Lesions
Source: Pathogens. 2024 May 16;13(5):416. doi: 10.3390/pathogens13050416 (PMC11124396; doi:10.3390/pathogens13050416)

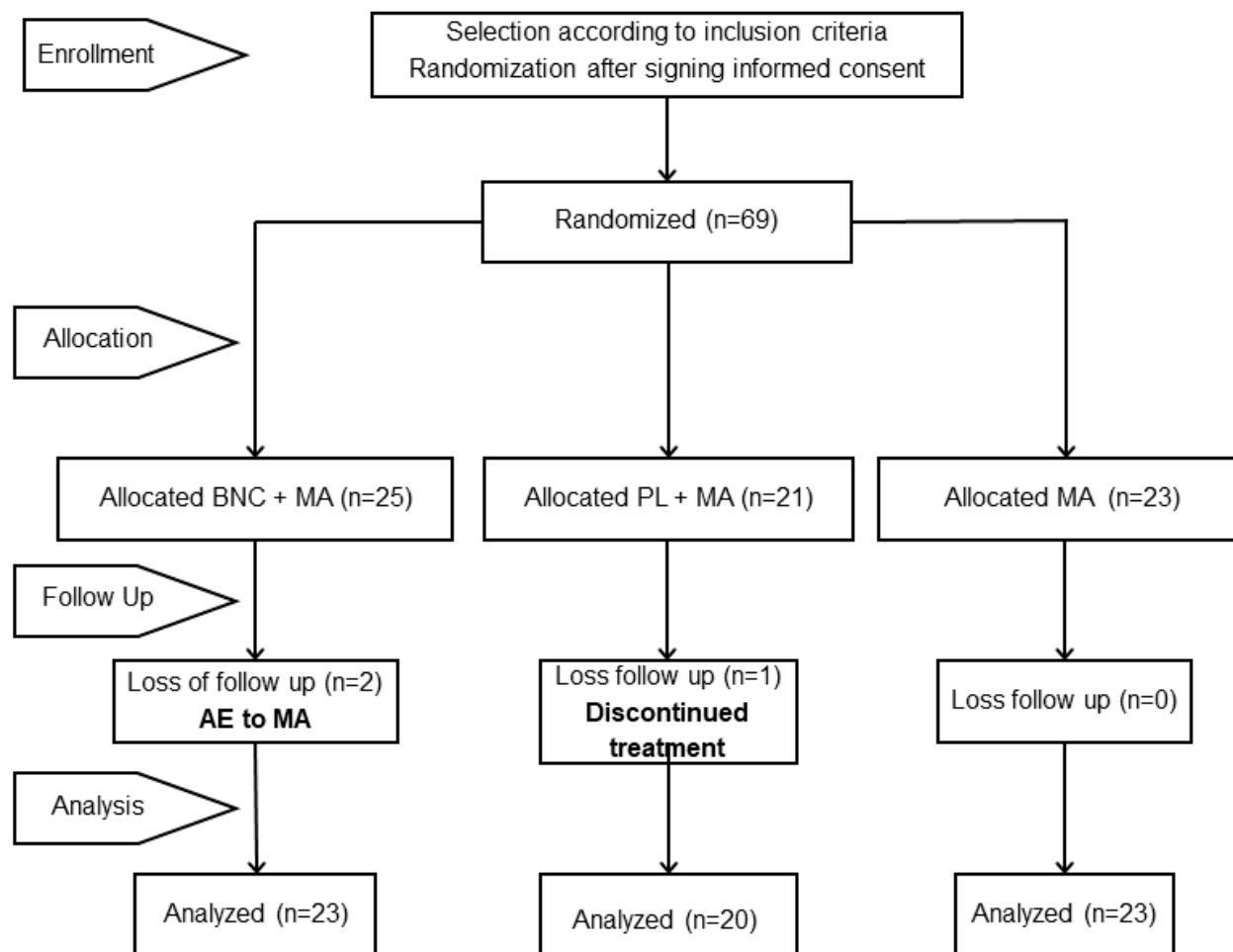

**Supplemental Figure S1.** Study flowchart.

Supplement: Supplementary file 1 [file pathogens-13-00416-s001.zip › Figure S1.pdf]
